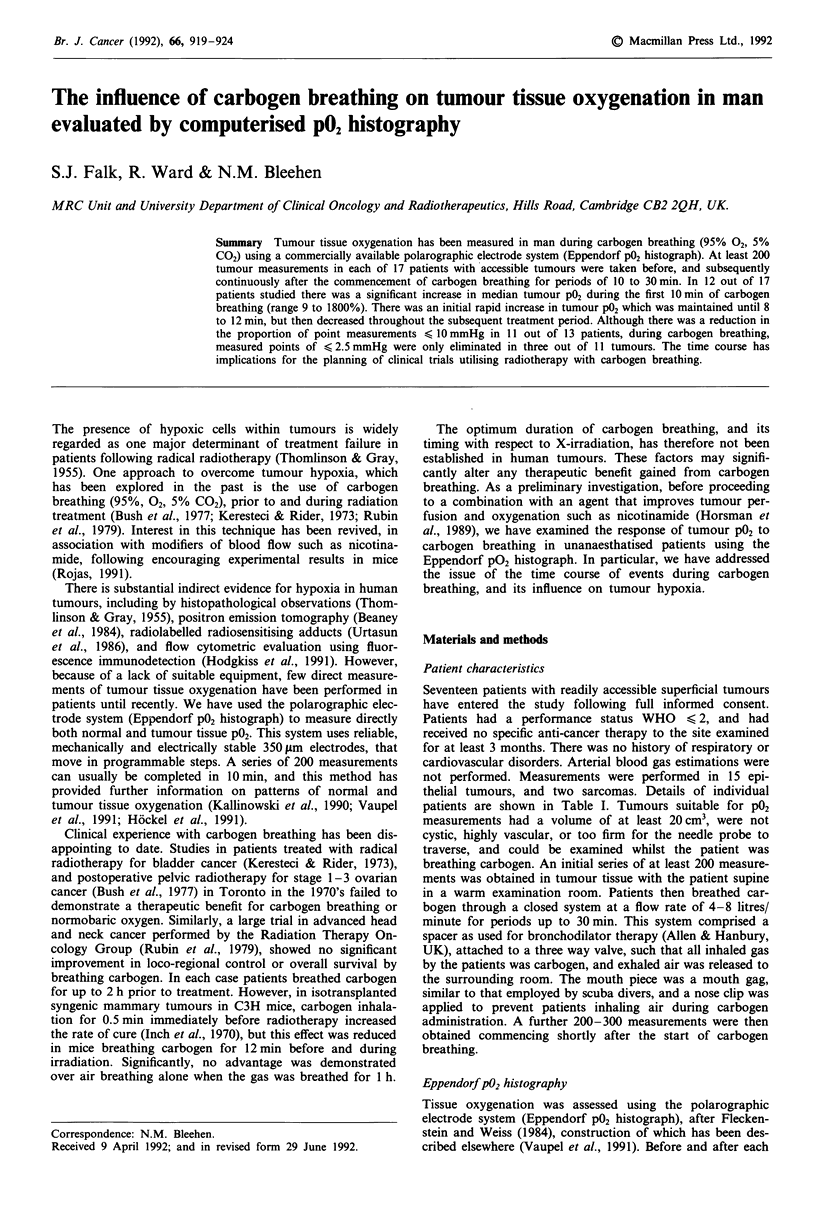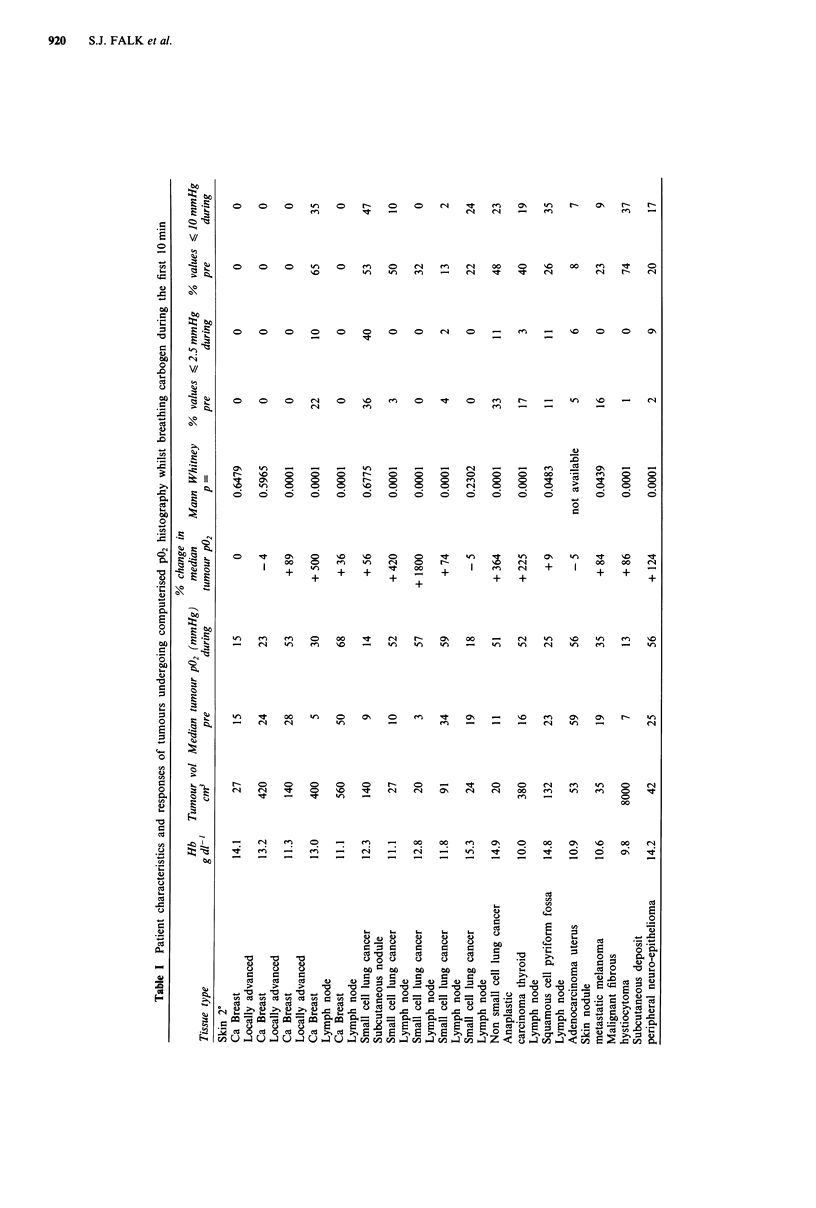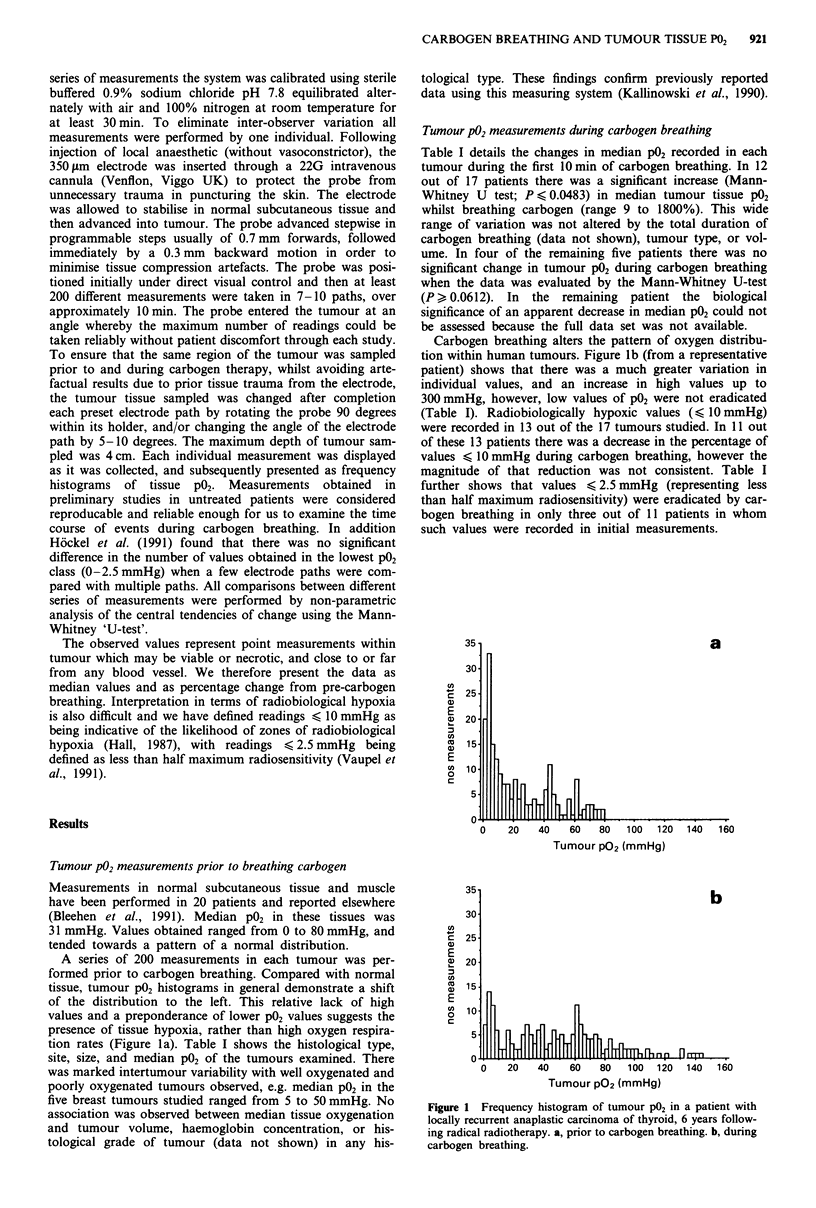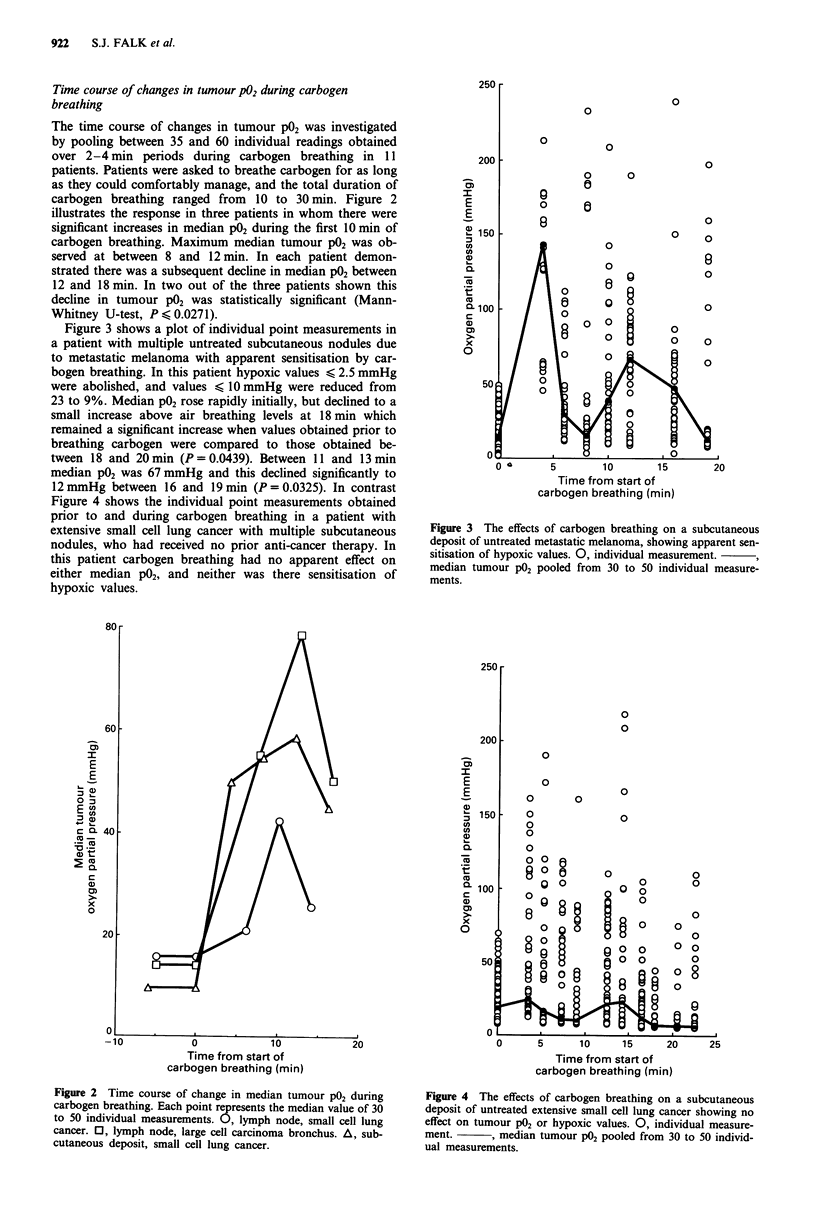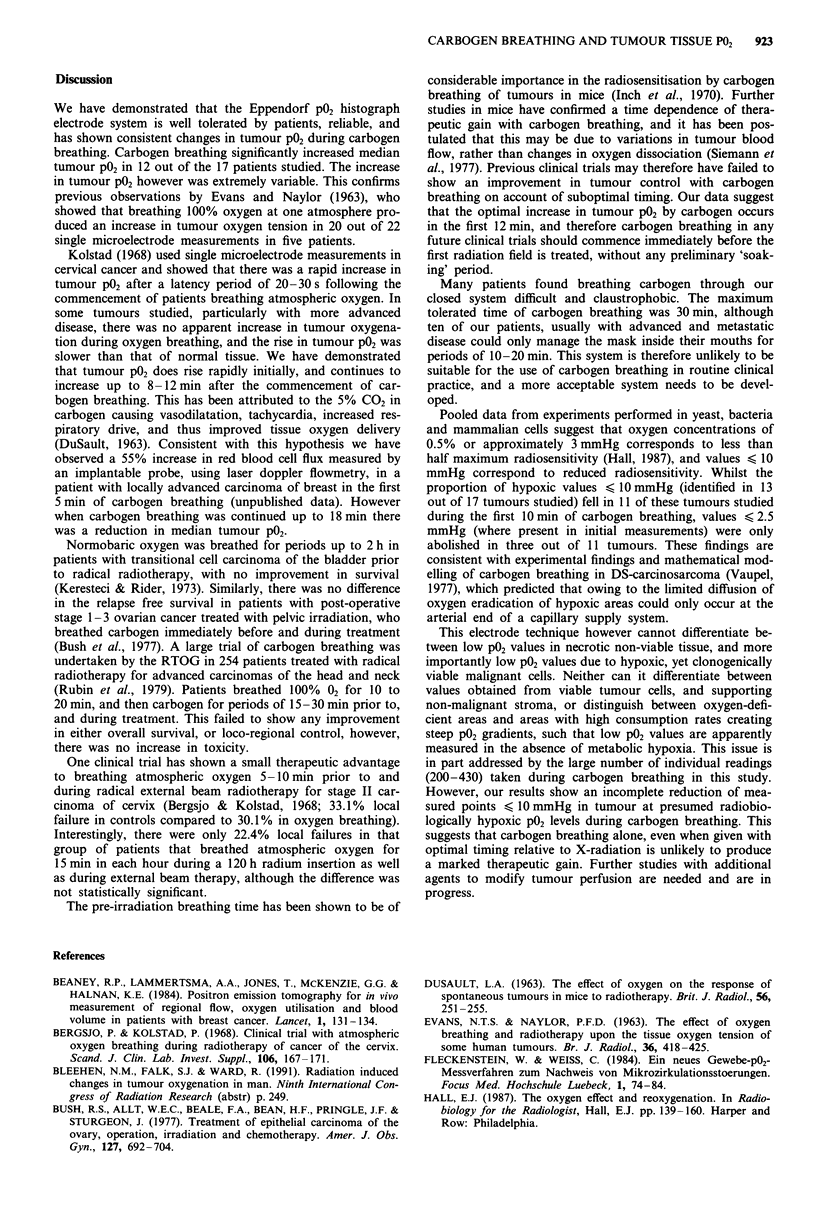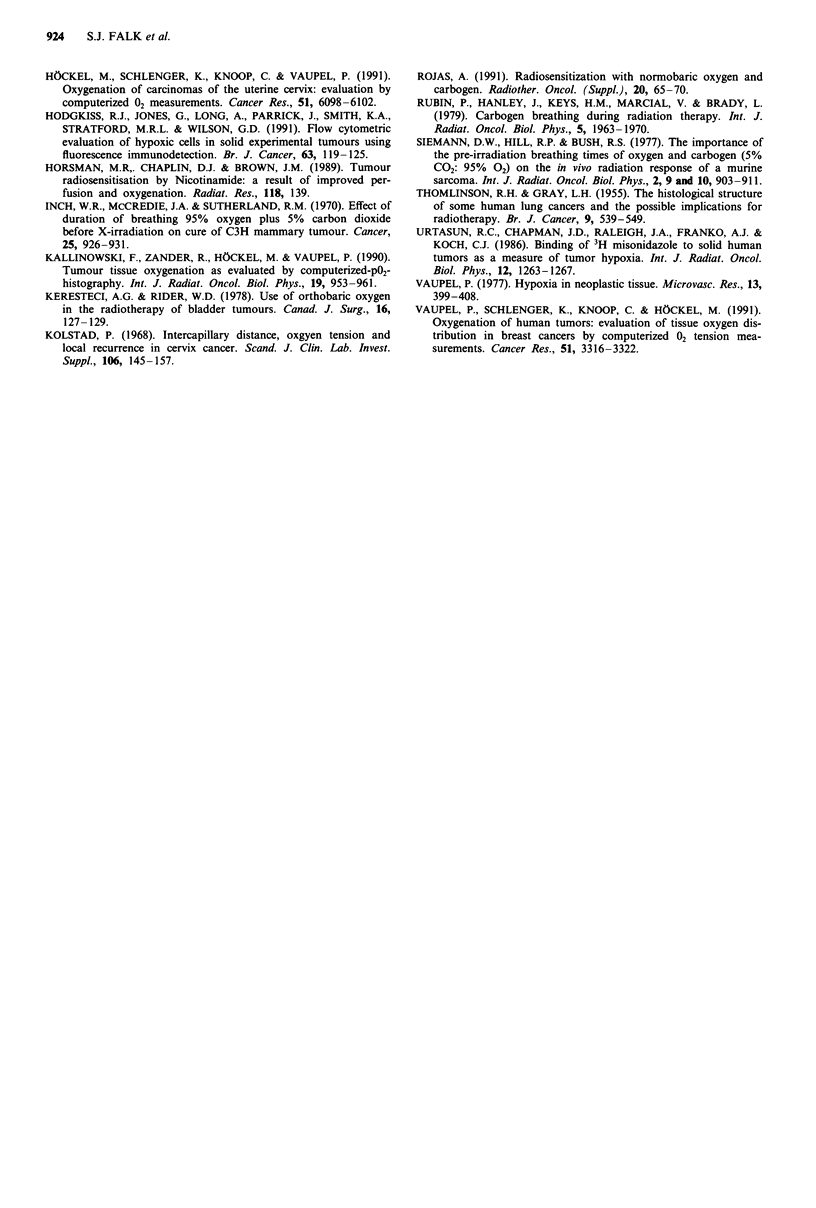# The influence of carbogen breathing on tumour tissue oxygenation in man evaluated by computerised p02 histography.

**DOI:** 10.1038/bjc.1992.386

**Published:** 1992-11

**Authors:** S. J. Falk, R. Ward, N. M. Bleehen

**Affiliations:** MRC Unit, Cambridge, UK.

## Abstract

Tumour tissue oxygenation has been measured in man during carbogen breathing (95% O2, 5% CO2) using a commercially available polarographic electrode system (Eppendorf p02 histograph). At least 200 tumour measurements in each of 17 patients with accessible tumours were taken before, and subsequently continuously after the commencement of carbogen breathing for periods of 10 to 30 min. In 12 out of 17 patients studied there was a significant increase in median tumour p02 during the first 10 min of carbogen breathing (range 9 to 1800%). There was an initial rapid increase in tumour p02 which was maintained until 8 to 12 min, but then decreased throughout the subsequent treatment period. Although there was a reduction in the proportion of point measurements < or = 10 mmHg in 11 out of 13 patients, during carbogen breathing, measured points of < or = 2.5 mmHg were only eliminated in three out of 11 tumours. The time course has implications for the planning of clinical trials utilising radiotherapy with carbogen breathing.


Br. J. Cancer (1992), 66, 919–924 © Macmillan Press Ltd., 1992

### The influence of carbogen breathing on tumour tissue oxygenation in man evaluated by computerised $pO_2$ histography

S.J. Falk, R. Ward & N.M. Bleehen

*MRC Unit and University Department of Clinical Oncology and Radiotherapeutics, Hills Road, Cambridge CB2 2QH, UK.*

**Summary** Tumour tissue oxygenation has been measured in man during carbogen breathing (95% $O_2$, 5% $CO_2$) using a commercially available polarographic electrode system (Eppendorf $pO_2$ histograph). At least 200 tumour measurements in each of 17 patients with accessible tumours were taken before, and subsequently continuously after the commencement of carbogen breathing for periods of 10 to 30 min. In 12 out of 17 patients studied there was a significant increase in median tumour $pO_2$ during the first 10 min of carbogen breathing (range 9 to 1800%). There was an initial rapid increase in tumour $pO_2$ which was maintained until 8 to 12 min, but then decreased throughout the subsequent treatment period. Although there was a reduction in the proportion of point measurements $\leqslant$ 10 mmHg in 11 out of 13 patients, during carbogen breathing, measured points of $\leqslant$ 2.5 mmHg were only eliminated in three out of 11 tumours. The time course has implications for the planning of clinical trials utilising radiotherapy with carbogen breathing.

The presence of hypoxic cells within tumours is widely regarded as one major determinant of treatment failure in patients following radical radiotherapy (Thomlinson & Gray, 1955). One approach to overcome tumour hypoxia, which has been explored in the past is the use of carbogen breathing (95%, $O_2$, 5% $CO_2$), prior to and during radiation treatment (Bush *et al.*, 1977; Keresteci & Rider, 1973; Rubin *et al.*, 1979). Interest in this technique has been revived, in association with modifiers of blood flow such as nicotinamide, following encouraging experimental results in mice (Rojas, 1991).

There is substantial indirect evidence for hypoxia in human tumours, including by histopathological observations (Thomlinson & Gray, 1955), positron emission tomography (Beaney *et al.*, 1984), radiolabelled radiosensitising adducts (Urtasun *et al.*, 1986), and flow cytometric evaluation using fluorescence immunodetection (Hodgkiss *et al.*, 1991). However, because of a lack of suitable equipment, few direct measurements of tumour tissue oxygenation have been performed in patients until recently. We have used the polarographic electrode system (Eppendorf $pO_2$ histograph) to measure directly both normal and tumour tissue $pO_2$. This system uses reliable, mechanically and electrically stable 350 $\mu$m electrodes, that move in programmable steps. A series of 200 measurements can usually be completed in 10 min, and this method has provided further information on patterns of normal and tumour tissue oxygenation (Kallinowski *et al.*, 1990; Vaupel *et al.*, 1991; Höckel *et al.*, 1991).

Clinical experience with carbogen breathing has been disappointing to date. Studies in patients treated with radical radiotherapy for bladder cancer (Keresteci & Rider, 1973), and postoperative pelvic radiotherapy for stage 1–3 ovarian cancer (Bush *et al.*, 1977) in Toronto in the 1970's failed to demonstrate a therapeutic benefit for carbogen breathing or normobaric oxygen. Similarly, a large trial in advanced head and neck cancer performed by the Radiation Therapy Oncology Group (Rubin *et al.*, 1979), showed no significant improvement in loco-regional control or overall survival by breathing carbogen. In each case patients breathed carbogen for up to 2 h prior to treatment. However, in isotransplanted syngenic mammary tumours in C3H mice, carbogen inhalation for 0.5 min immediately before radiotherapy increased the rate of cure (Inch *et al.*, 1970), but this effect was reduced in mice breathing carbogen for 12 min before and during irradiation. Significantly, no advantage was demonstrated over air breathing alone when the gas was breathed for 1 h.

The optimum duration of carbogen breathing, and its timing with respect to X-irradiation, has therefore not been established in human tumours. These factors may significantly alter any therapeutic benefit gained from carbogen breathing. As a preliminary investigation, before proceeding to a combination with an agent that improves tumour perfusion and oxygenation such as nicotinamide (Horsman *et al.*, 1989), we have examined the response of tumour $pO_2$ to carbogen breathing in unanaesthatised patients using the Eppendorf $pO_2$ histograph. In particular, we have addressed the issue of the time course of events during carbogen breathing, and its influence on tumour hypoxia.

### Materials and methods

### *Patient characteristics*

Seventeen patients with readily accessible superficial tumours have entered the study following full informed consent. Patients had a performance status WHO $\leqslant 2$, and had received no specific anti-cancer therapy to the site examined for at least 3 months. There was no history of respiratory or cardiovascular disorders. Arterial blood gas estimations were not performed. Measurements were performed in 15 epithelial tumours, and two sarcomas. Details of individual patients are shown in Table I. Tumours suitable for $pO_2$ measurements had a volume of at least 20 $cm^3$, were not cystic, highly vascular, or too firm for the needle probe to traverse, and could be examined whilst the patient was breathing carbogen. An initial series of at least 200 measurements was obtained in tumour tissue with the patient supine in a warm examination room. Patients then breathed carbogen through a closed system at a flow rate of 4–8 litres/minute for periods up to 30 min. This system comprised a spacer as used for bronchodilator therapy (Allen & Hanbury, UK), attached to a three way valve, such that all inhaled gas by the patients was carbogen, and exhaled air was released to the surrounding room. The mouth piece was a mouth gag, similar to that employed by scuba divers, and a nose clip was applied to prevent patients inhaling air during carbogen administration. A further 200–300 measurements were then obtained commencing shortly after the start of carbogen breathing.

### *Eppendorf $pO_2$ histography*

Tissue oxygenation was assessed using the polarographic electrode system (Eppendorf $pO_2$ histograph), after Fleckenstein and Weiss (1984), construction of which has been described elsewhere (Vaupel *et al.*, 1991). Before and after each

Correspondence: N.M. Bleehen.
Received 9 April 1992; and in revised form 29 June 1992.

**Table I** Patient characteristics and responses of tumours undergoing computerised $pO_2$ histography whilst breathing carbogen during the first 10 min

| *Tissue type* | *Hb* $g\,dl^{-1}$ | *Tumour vol* $cm^3$ | *Median tumour* $pO_2$ *(mmHg)* *pre* | *during* | *% change in median tumour* $pO_2$ | *Mann Whitney* $p=$ | *% values* $\leqslant 2.5$ *mmHg* *pre* | *during* | *% values* $\leqslant 10$ *mmHg* *pre* | *during* |
|---|---|---|---|---|---|---|---|---|---|---|
| Skin 2° Ca Breast | 14.1 | 27 | 15 | 15 | 0 | 0.6479 | 0 | 0 | 0 | 0 |
| Locally advanced Ca Breast | 13.2 | 420 | 24 | 23 | − 4 | 0.5965 | 0 | 0 | 0 | 0 |
| Locally advanced Ca Breast | 11.3 | 140 | 28 | 53 | + 89 | 0.0001 | 0 | 0 | 0 | 0 |
| Locally advanced Ca Breast | 13.0 | 400 | 5 | 30 | + 500 | 0.0001 | 22 | 10 | 65 | 35 |
| Lymph node Ca Breast | 11.1 | 560 | 50 | 68 | + 36 | 0.0001 | 0 | 0 | 0 | 0 |
| Lymph node Small cell lung cancer | 12.3 | 140 | 9 | 14 | + 56 | 0.6775 | 36 | 40 | 53 | 47 |
| Subcutaneous nodule Small cell lung cancer | 11.1 | 27 | 10 | 52 | + 420 | 0.0001 | 3 | 0 | 50 | 10 |
| Lymph node Small cell lung cancer | 12.8 | 20 | 3 | 57 | + 1800 | 0.0001 | 0 | 0 | 32 | 0 |
| Lymph node Small cell lung cancer | 11.8 | 91 | 34 | 59 | + 74 | 0.0001 | 4 | 2 | 13 | 2 |
| Lymph node Small cell lung cancer | 15.3 | 24 | 19 | 18 | − 5 | 0.2302 | 0 | 0 | 22 | 24 |
| Lymph node Non small cell lung cancer | 14.9 | 20 | 11 | 51 | + 364 | 0.0001 | 33 | 11 | 48 | 23 |
| Anaplastic carcinoma thyroid | 10.0 | 380 | 16 | 52 | + 225 | 0.0001 | 17 | 3 | 40 | 19 |
| Lymph node Squamous cell pyriform fossa | 14.8 | 132 | 23 | 25 | + 9 | 0.0483 | 11 | 11 | 26 | 35 |
| Lymph node Adenocarcinoma uterus | 10.9 | 53 | 59 | 56 | − 5 | not available | 5 | 6 | 8 | 7 |
| Skin nodule metastatic melanoma | 10.6 | 35 | 19 | 35 | + 84 | 0.0439 | 16 | 0 | 23 | 9 |
| Malignant fibrous hystiocytoma | 9.8 | 8000 | 7 | 13 | + 86 | 0.0001 | 1 | 0 | 74 | 37 |
| Subcutaneous deposit peripheral neuro-epithelioma | 14.2 | 42 | 25 | 56 | + 124 | 0.0001 | 2 | 9 | 20 | 17 |

series of measurements the system was calibrated using sterile buffered 0.9% sodium chloride pH 7.8 equilibrated alternately with air and 100% nitrogen at room temperature for at least 30 min. To eliminate inter-observer variation all measurements were performed by one individual. Following injection of local anaesthetic (without vasoconstrictor), the 350 $\mu$m electrode was inserted through a 22G intravenous cannula (Venflon, Viggo UK) to protect the probe from unnecessary trauma in puncturing the skin. The electrode was allowed to stabilise in normal subcutaneous tissue and then advanced into tumour. The probe advanced stepwise in programmable steps usually of 0.7 mm forwards, followed immediately by a 0.3 mm backward motion in order to minimise tissue compression artefacts. The probe was positioned initially under direct visual control and then at least 200 different measurements were taken in 7–10 paths, over approximately 10 min. The probe entered the tumour at an angle whereby the maximum number of readings could be taken reliably without patient discomfort through each study. To ensure that the same region of the tumour was sampled prior to and during carbogen therapy, whilst avoiding artefactual results due to prior tissue trauma from the electrode, the tumour tissue sampled was changed after completion each preset electrode path by rotating the probe 90 degrees within its holder, and/or changing the angle of the electrode path by 5–10 degrees. The maximum depth of tumour sampled was 4 cm. Each individual measurement was displayed as it was collected, and subsequently presented as frequency histograms of tissue $p0_2$. Measurements obtained in preliminary studies in untreated patients were considered reproducable and reliable enough for us to examine the time course of events during carbogen breathing. In addition Höckel *et al.* (1991) found that there was no significant difference in the number of values obtained in the lowest $p0_2$ class (0–2.5 mmHg) when a few electrode paths were compared with multiple paths. All comparisons between different series of measurements were performed by non-parametric analysis of the central tendencies of change using the Mann-Whitney 'U-test'.

The observed values represent point measurements within tumour which may be viable or necrotic, and close to or far from any blood vessel. We therefore present the data as median values and as percentage change from pre-carbogen breathing. Interpretation in terms of radiobiological hypoxia is also difficult and we have defined readings $\leqslant$ 10 mmHg as being indicative of the likelihood of zones of radiobiological hypoxia (Hall, 1987), with readings $\leqslant$ 2.5 mmHg being defined as less than half maximum radiosensitivity (Vaupel *et al.*, 1991).

### Results

### *Tumour $p0_2$ measurements prior to breathing carbogen*

Measurements in normal subcutaneous tissue and muscle have been performed in 20 patients and reported elsewhere (Bleehen *et al.*, 1991). Median $p0_2$ in these tissues was 31 mmHg. Values obtained ranged from 0 to 80 mmHg, and tended towards a pattern of a normal distribution.

A series of 200 measurements in each tumour was performed prior to carbogen breathing. Compared with normal tissue, tumour $p0_2$ histograms in general demonstrate a shift of the distribution to the left. This relative lack of high values and a preponderance of lower $p0_2$ values suggests the presence of tissue hypoxia, rather than high oxygen respiration rates (Figure 1a). Table I shows the histological type, site, size, and median $p0_2$ of the tumours examined. There was marked intertumour variability with well oxygenated and poorly oxygenated tumours observed, e.g. median $p0_2$ in the five breast tumours studied ranged from 5 to 50 mmHg. No association was observed between median tissue oxygenation and tumour volume, haemoglobin concentration, or histological grade of tumour (data not shown) in any histological type. These findings confirm previously reported data using this measuring system (Kallinowski *et al.*, 1990).

### *Tumour $p0_2$ measurements during carbogen breathing*

Table I details the changes in median $p0_2$ recorded in each tumour during the first 10 min of carbogen breathing. In 12 out of 17 patients there was a significant increase (Mann-Whitney U test; $P \leqslant 0.0483$) in median tumour tissue $p0_2$ whilst breathing carbogen (range 9 to 1800%). This wide range of variation was not altered by the total duration of carbogen breathing (data not shown), tumour type, or volume. In four of the remaining five patients there was no significant change in tumour $p0_2$ during carbogen breathing when the data was evaluated by the Mann-Whitney U-test ($P \geqslant 0.0612$). In the remaining patient the biological significance of an apparent decrease in median $p0_2$ could not be assessed because the full data set was not available.

Carbogen breathing alters the pattern of oxygen distribution within human tumours. Figure 1b (from a representative patient) shows that there was a much greater variation in individual values, and an increase in high values up to 300 mmHg, however, low values of $p0_2$ were not eradicated (Table I). Radiobiologically hypoxic values ($\leqslant$ 10 mmHg) were recorded in 13 out of the 17 tumours studied. In 11 out of these 13 patients there was a decrease in the percentage of values $\leqslant$ 10 mmHg during carbogen breathing, however the magnitude of that reduction was not consistent. Table I further shows that values $\leqslant$ 2.5 mmHg (representing less than half maximum radiosensitivity) were eradicated by carbogen breathing in only three out of 11 patients in whom such values were recorded in initial measurements.

**Figure 1** Frequency histogram of tumour $p0_2$ in a patient with locally recurrent anaplastic carcinoma of thyroid, 6 years following radical radiotherapy. **a**, prior to carbogen breathing. **b**, during carbogen breathing.

*Time course of changes in tumour $pO_2$ during carbogen breathing*

The time course of changes in tumour $pO_2$ was investigated by pooling between 35 and 60 individual readings obtained over 2–4 min periods during carbogen breathing in 11 patients. Patients were asked to breathe carbogen for as long as they could comfortably manage, and the total duration of carbogen breathing ranged from 10 to 30 min. Figure 2 illustrates the response in three patients in whom there were significant increases in median $pO_2$ during the first 10 min of carbogen breathing. Maximum median tumour $pO_2$ was observed at between 8 and 12 min. In each patient demonstrated there was a subsequent decline in median $pO_2$ between 12 and 18 min. In two out of the three patients shown this decline in tumour $pO_2$ was statistically significant (Mann-Whitney U-test, $P \leqslant 0.0271$).

Figure 3 shows a plot of individual point measurements in a patient with multiple untreated subcutaneous nodules due to metastatic melanoma with apparent sensitisation by carbogen breathing. In this patient hypoxic values $\leqslant 2.5$ mmHg were abolished, and values $\leqslant 10$ mmHg were reduced from 23 to 9%. Median $pO_2$ rose rapidly initially, but declined to a small increase above air breathing levels at 18 min which remained a significant increase when values obtained prior to breathing carbogen were compared to those obtained between 18 and 20 min ($P = 0.0439$). Between 11 and 13 min median $pO_2$ was 67 mmHg and this declined significantly to 12 mmHg between 16 and 19 min ($P = 0.0325$). In contrast Figure 4 shows the individual point measurements obtained prior to and during carbogen breathing in a patient with extensive small cell lung cancer with multiple subcutaneous nodules, who had received no prior anti-cancer therapy. In this patient carbogen breathing had no apparent effect on either median $pO_2$, and neither was there sensitisation of hypoxic values.

**Figure 3** The effects of carbogen breathing on a subcutaneous deposit of untreated metastatic melanoma, showing apparent sensitisation of hypoxic values. O, individual measurement. ———, median tumour $pO_2$ pooled from 30 to 50 individual measurements.

**Figure 2** Time course of change in median tumour $pO_2$ during carbogen breathing. Each point represents the median value of 30 to 50 individual measurements. O, lymph node, small cell lung cancer. □, lymph node, large cell carcinoma bronchus. Δ, subcutaneous deposit, small cell lung cancer.

**Figure 4** The effects of carbogen breathing on a subcutaneous deposit of untreated extensive small cell lung cancer showing no effect on tumour $pO_2$ or hypoxic values. O, individual measurement. ———, median tumour $pO_2$ pooled from 30 to 50 individual measurements.

### Discussion

We have demonstrated that the Eppendorf $p0_2$ histograph electrode system is well tolerated by patients, reliable, and has shown consistent changes in tumour $p0_2$ during carbogen breathing. Carbogen breathing significantly increased median tumour $p0_2$ in 12 out of the 17 patients studied. The increase in tumour $p0_2$ however was extremely variable. This confirms previous observations by Evans and Naylor (1963), who showed that breathing 100% oxygen at one atmosphere produced an increase in tumour oxygen tension in 20 out of 22 single microelectrode measurements in five patients.

Kolstad (1968) used single microelectrode measurements in cervical cancer and showed that there was a rapid increase in tumour $p0_2$ after a latency period of 20–30 s following the commencement of patients breathing atmospheric oxygen. In some tumours studied, particularly with more advanced disease, there was no apparent increase in tumour oxygenation during oxygen breathing, and the rise in tumour $p0_2$ was slower than that of normal tissue. We have demonstrated that tumour $p0_2$ does rise rapidly initially, and continues to increase up to 8–12 min after the commencement of carbogen breathing. This has been attributed to the 5% $CO_2$ in carbogen causing vasodilatation, tachycardia, increased respiratory drive, and thus improved tissue oxygen delivery (DuSault, 1963). Consistent with this hypothesis we have observed a 55% increase in red blood cell flux measured by an implantable probe, using laser doppler flowmetry, in a patient with locally advanced carcinoma of breast in the first 5 min of carbogen breathing (unpublished data). However when carbogen breathing was continued up to 18 min there was a reduction in median tumour $p0_2$.

Normobaric oxygen was breathed for periods up to 2 h in patients with transitional cell carcinoma of the bladder prior to radical radiotherapy, with no improvement in survival (Keresteci & Rider, 1973). Similarly, there was no difference in the relapse free survival in patients with post-operative stage 1–3 ovarian cancer treated with pelvic irradiation, who breathed carbogen immediately before and during treatment (Bush *et al.*, 1977). A large trial of carbogen breathing was undertaken by the RTOG in 254 patients treated with radical radiotherapy for advanced carcinomas of the head and neck (Rubin *et al.*, 1979). Patients breathed 100% $0_2$ for 10 to 20 min, and then carbogen for periods of 15–30 min prior to, and during treatment. This failed to show any improvement in either overall survival, or loco-regional control, however, there was no increase in toxicity.

One clinical trial has shown a small therapeutic advantage to breathing atmospheric oxygen 5–10 min prior to and during radical external beam radiotherapy for stage II carcinoma of cervix (Bergsjo & Kolstad, 1968; 33.1% local failure in controls compared to 30.1% in oxygen breathing). Interestingly, there were only 22.4% local failures in that group of patients that breathed atmospheric oxygen for 15 min in each hour during a 120 h radium insertion as well as during external beam therapy, although the difference was not statistically significant.

The pre-irradiation breathing time has been shown to be of considerable importance in the radiosensitisation by carbogen breathing of tumours in mice (Inch *et al.*, 1970). Further studies in mice have confirmed a time dependence of therapeutic gain with carbogen breathing, and it has been postulated that this may be due to variations in tumour blood flow, rather than changes in oxygen dissociation (Siemann *et al.*, 1977). Previous clinical trials may therefore have failed to show an improvement in tumour control with carbogen breathing on account of suboptimal timing. Our data suggest that the optimal increase in tumour $p0_2$ by carbogen occurs in the first 12 min, and therefore carbogen breathing in any future clinical trials should commence immediately before the first radiation field is treated, without any preliminary 'soaking' period.

Many patients found breathing carbogen through our closed system difficult and claustrophobic. The maximum tolerated time of carbogen breathing was 30 min, although ten of our patients, usually with advanced and metastatic disease could only manage the mask inside their mouths for periods of 10–20 min. This system is therefore unlikely to be suitable for the use of carbogen breathing in routine clinical practice, and a more acceptable system needs to be developed.

Pooled data from experiments performed in yeast, bacteria and mammalian cells suggest that oxygen concentrations of 0.5% or approximately 3 mmHg corresponds to less than half maximum radiosensitivity (Hall, 1987), and values $\leqslant 10$ mmHg correspond to reduced radiosensitivity. Whilst the proportion of hypoxic values $\leqslant 10$ mmHg (identified in 13 out of 17 tumours studied) fell in 11 of these tumours studied during the first 10 min of carbogen breathing, values $\leqslant 2.5$ mmHg (where present in initial measurements) were only abolished in three out of 11 tumours. These findings are consistent with experimental findings and mathematical modelling of carbogen breathing in DS-carcinosarcoma (Vaupel, 1977), which predicted that owing to the limited diffusion of oxygen eradication of hypoxic areas could only occur at the arterial end of a capillary supply system.

This electrode technique however cannot differentiate between low $p0_2$ values in necrotic non-viable tissue, and more importantly low $p0_2$ values due to hypoxic, yet clonogenically viable malignant cells. Neither can it differentiate between values obtained from viable tumour cells, and supporting non-malignant stroma, or distinguish between oxygen-deficient areas and areas with high consumption rates creating steep $p0_2$ gradients, such that low $p0_2$ values are apparently measured in the absence of metabolic hypoxia. This issue is in part addressed by the large number of individual readings (200–430) taken during carbogen breathing in this study. However, our results show an incomplete reduction of measured points $\leqslant 10$ mmHg in tumour at presumed radiobiologically hypoxic $p0_2$ levels during carbogen breathing. This suggests that carbogen breathing alone, even when given with optimal timing relative to X-radiation is unlikely to produce a marked therapeutic gain. Further studies with additional agents to modify tumour perfusion are needed and are in progress.

### References

BEANEY, R.P., LAMMERTSMA, A.A., JONES, T., MCKENZIE, G.G. & HALNAN, K.E. (1984). Positron emission tomography for *in vivo* measurement of regional flow, oxygen utilisation and blood volume in patients with breast cancer. *Lancet*, **1**, 131–134.

BERGSJO, P. & KOLSTAD, P. (1968). Clinical trial with atmospheric oxygen breathing during radiotherapy of cancer of the cervix. *Scand. J. Clin. Lab. Invest. Suppl.*, **106**, 167–171.

BLEEHEN, N.M., FALK, S.J. & WARD, R. (1991). Radiation induced changes in tumour oxygenation in man. *Ninth International Congress of Radiation Research* (abstr) p. 249.

BUSH, R.S., ALLT, W.E.C., BEALE, F.A., BEAN, H.F., PRINGLE, J.F. & STURGEON, J. (1977). Treatment of epithelial carcinoma of the ovary, operation, irradiation and chemotherapy. *Amer. J. Obs. Gyn.*, **127**, 692–704.

DUSAULT, L.A. (1963). The effect of oxygen on the response of spontaneous tumours in mice to radiotherapy. *Brit. J. Radiol.*, **56**, 251–255.

EVANS, N.T.S. & NAYLOR, P.F.D. (1963). The effect of oxygen breathing and radiotherapy upon the tissue oxygen tension of some human tumours. *Br. J. Radiol.*, **36**, 418–425.

FLECKENSTEIN, W. & WEISS, C. (1984). Ein neues Gewebe-$p0_2$-Messverfahren zum Nachweis von Mikrozirkulationsstoerungen. *Focus Med. Hochschule Luebeck*, **1**, 74–84.

HALL, E.J. (1987). The oxygen effect and reoxygenation. In *Radiobiology for the Radiologist*, Hall, E.J. pp. 139–160. Harper and Row: Philadelphia.

HÖCKEL, M., SCHLENGER, K., KNOOP, C. & VAUPEL, P. (1991). Oxygenation of carcinomas of the uterine cervix: evaluation by computerized $0_2$ measurements. *Cancer Res.*, **51**, 6098–6102.

HODGKISS, R.J., JONES, G., LONG, A., PARRICK, J., SMITH, K.A., STRATFORD, M.R.L. & WILSON, G.D. (1991). Flow cytometric evaluation of hypoxic cells in solid experimental tumours using fluorescence immunodetection. *Br. J. Cancer*, **63**, 119–125.

HORSMAN, M.R,. CHAPLIN, D.J. & BROWN, J.M. (1989). Tumour radiosensitisation by Nicotinamide: a result of improved perfusion and oxygenation. *Radiat. Res.*, **118**, 139.

INCH, W.R., MCCREDIE, J.A. & SUTHERLAND, R.M. (1970). Effect of duration of breathing 95% oxygen plus 5% carbon dioxide before X-irradiation on cure of C3H mammary tumour. *Cancer*, **25**, 926–931.

KALLINOWSKI, F., ZANDER, R., HÖCKEL, M. & VAUPEL, P. (1990). Tumour tissue oxygenation as evaluated by computerized-$p0_2$-histography. *Int. J. Radiat. Oncol. Biol. Phys.*, **19**, 953–961.

KERESTECI, A.G. & RIDER, W.D. (1978). Use of orthobaric oxygen in the radiotherapy of bladder tumours. *Canad. J. Surg.*, **16**, 127–129.

KOLSTAD, P. (1968). Intercapillary distance, oxgyen tension and local recurrence in cervix cancer. *Scand. J. Clin. Lab. Invest. Suppl.*, **106**, 145–157.

ROJAS, A. (1991). Radiosensitization with normobaric oxygen and carbogen. *Radiother. Oncol. (Suppl.)*, **20**, 65–70.

RUBIN, P., HANLEY, J., KEYS, H.M., MARCIAL, V. & BRADY, L. (1979). Carbogen breathing during radiation therapy. *Int. J. Radiat. Oncol. Biol. Phys.*, **5**, 1963–1970.

SIEMANN, D.W., HILL, R.P. & BUSH, R.S. (1977). The importance of the pre-irradiation breathing times of oxygen and carbogen (5% $CO_2$: 95% $O_2$) on the *in vivo* radiation response of a murine sarcoma. *Int. J. Radiat. Oncol. Biol. Phys.*, **2, 9 and 10,** 903–911.

THOMLINSON, R.H. & GRAY, L.H. (1955). The histological structure of some human lung cancers and the possible implications for radiotherapy. *Br. J. Cancer*, **9**, 539–549.

URTASUN, R.C., CHAPMAN, J.D., RALEIGH, J.A., FRANKO, A.J. & KOCH, C.J. (1986). Binding of $^3H$ misonidazole to solid human tumors as a measure of tumor hypoxia. *Int. J. Radiat. Oncol. Biol. Phys.*, **12**, 1263–1267.

VAUPEL, P. (1977). Hypoxia in neoplastic tissue. *Microvasc. Res.*, **13**, 399–408.

VAUPEL, P., SCHLENGER, K., KNOOP, C. & HÖCKEL, M. (1991). Oxygenation of human tumors: evaluation of tissue oxygen distribution in breast cancers by computerized $0_2$ tension measurements. *Cancer Res.*, **51**, 3316–3322.